# The ClC Cl^–^ channel CLH-1 mediates HCO_3_^–^ efflux from the amphid sheath glia in *C. elegans*

**DOI:** 10.17912/micropub.biology.000510

**Published:** 2022-01-12

**Authors:** Jesus Fernandez-Abascal, Laura Bianchi

**Affiliations:** 1 Department of Physiology and Biophysics, Miller School of Medicine, University of Miami, 1600 NW 10th Ave, Mimi, FL, USA

## Abstract

Cellular function is regulated by the concentration of intracellular and extracellular ions, including pH. Ion channels and transporters that mediate the flux/transport of protons and bicarbonate (HCO_3_^–^) are the chief regulators of pH. In the nervous system, due to their high electrical activity, neurons tend to produce and excrete large amounts of acids. On the contrary, glial cells have been proposed to be key contributors of pH buffering. We published that the Cl^–^/HCO_3_^–^ permeable channel CLH-1 mediates intracellular pH buffering of *C. elegans* Amphid sheath (AMsh) glia at baseline. We show here that, under physiological conditions, *clh-1 *knock out worms show reduced HCO_3_^– ^extrusion from AMsh glia, suggesting that CLH-1 may help prevent cellular alkalinization. This function becomes even more apparent when animals are grown on plates enriched with HCO_3_^–^. We speculate that CLH-1 might function as a regulator of extracellular pH.

**Figure 1. Cl- channel CLH-1 is needed in AMsh glia for HCO3- efflux:**
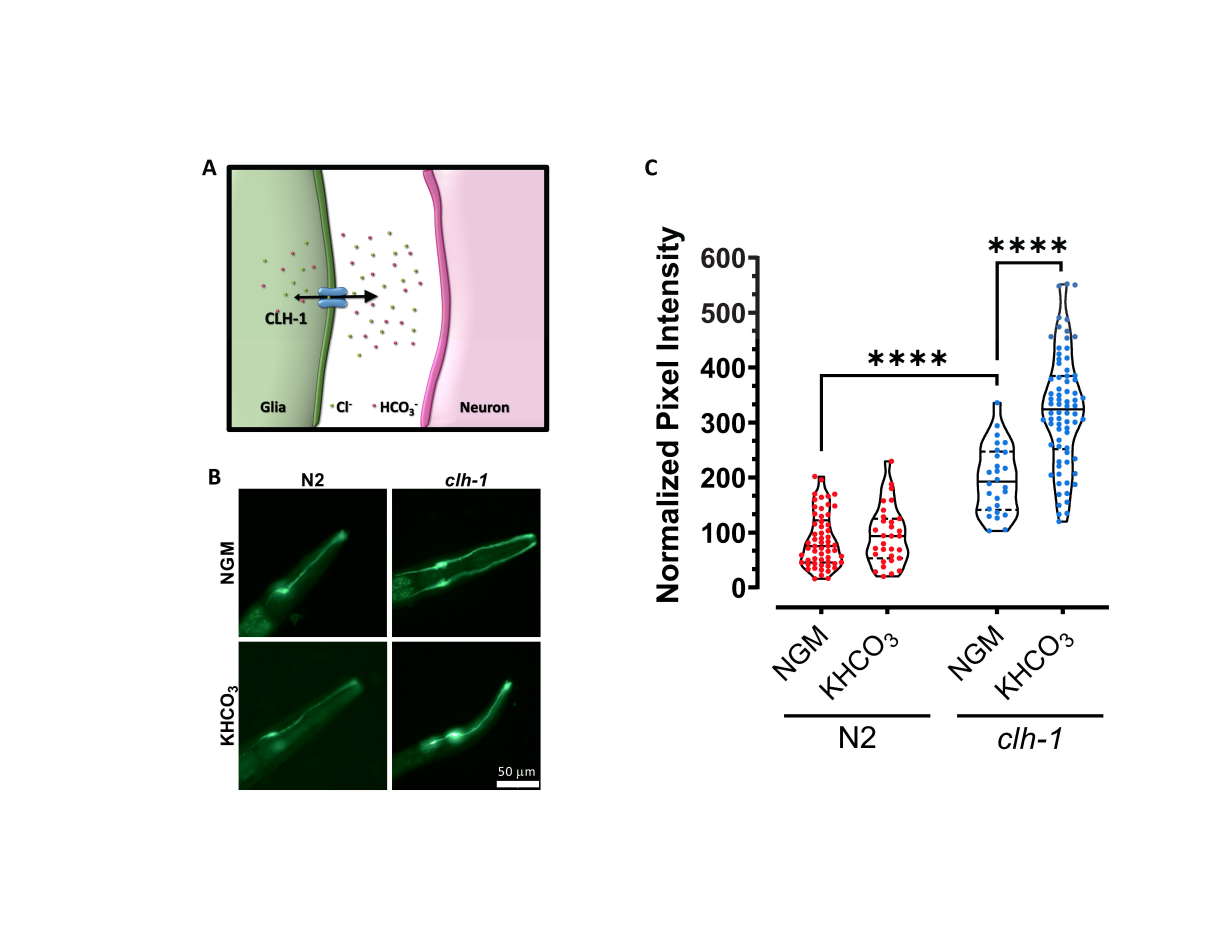
(A) Schematic representation of the chloride channel CLH-1 in glia and its function. CLH-1 mediates both efflux (big arrowhead) and to a lesser extent influx (small arrowhead) of Cl^–^ and HCO_3_^–^ thereby regulating the concentration of Cl^–^ and pH both inside and outside of the cell (Grant *et al.*, 2015, Fernandez-Abascal *et al.*, 2021). (B) Representative images of wild type (N2, left column) and *clh-1(ok658)* mutants (*clh-1*, right column) expressing the pH sensor Superecliptic pHluorin (pGM87) under the control of the AMsh glia specific promoter *pT02B11.3*, grown in normal conditions (NGM, top row) or in NGM plates supplemented with 150 mM KHCO_3 _(bottom row). Scale bar is 50 µm. (C) Violin plots showing the quantification of relative pH inside the AMsh glia as pixel intensity of pGM87 in wild type (red) and *clh-1(ok658)* mutants (blue) grown on NGM or NGM supplemented with KHCO_3 _plates_._ Data were normalized to N2 in NGM conditions. Each data point represents one worm (n= 58, 31, 26 and 74, respectively). Statistics were done by ANOVA followed by Tukey post-test (****p<0.0001).

## Description

Cellular function is regulated by both intracellular and extracellular pH. Proton and bicarbonate transporters and exchangers, as well as ion channels permeable to protons and bicarbonate (HCO_3_^–^) participate to pH regulation (Chesler, 2003). Importantly, channels and transporters that regulate extracellular pH have the potential of influencing the function of nearby cells, making them particularly critical for the function of a tissue or organ as a whole. Extracellular pH regulation, and in particular buffering of extracellular acid loads, is especially important in the nervous system where high neuronal activity leads to the production and release in the extracellular space of a significant amount of acid.

We published that the ClC Cl^–^ channel CLH-1 is expressed in the Amphid sheath glial cells (AMsh glia) of the *C. elegans* amphid apparatus (Grant *et al.*, 2015). These glial cells enwrap the sensory dendrites of 12 pairs of sensory neurons and are important for the structure and the function of these neurons (Bacaj *et al.*, 2008, Singhvi *et al.*, 2016, Raiders *et al.*, 2021, Wang *et al.*, 2008, Wang *et al.*, 2012, Fernandez-Abascal *et al.*, 2021, Wallace *et al.*, 2016, Oikonomou *et al.*, 2011, Razzauti and Laurent, 2021). Using the genetically encoded pH indicator Superecliptic pHlourin, we showed that CLH-1 is needed for influx of HCO_3_^–^ into the AMsh glia at baseline pH, following removal of HCO_3_^– ^from the extracellular space (Grant *et al.*, 2015). However, by electrophysiological analysis of CLH-1 ionic currents in *Xenopus* oocytes and mammalian HEK cells, we also showed that this channel conducts ions preferably from the inside to the outside of the cell (Grant *et al.*, 2015). This finding suggests that CLH-1 might mediate HCO_3_^–^ efflux *in vivo* thereby resulting in regulation of the pH outside of AMsh glia, potentially even in the microenvironment between AMsh glia and sensory dendrites (Fig. 1A). Consistent with CLH-1-mediated efflux of ions, we found that CLH-1 mediates Cl^–^ efflux from AMsh glia needed for GABA regulation of ASH neurons’ excitability upon nose touch stimulation (Fernandez-Abascal *et al.*, 2021).

To determine whether CLH-1 mediates HCO_3_^–^ efflux *in vivo*, we compared pHlourin fluorescence in AMsh glia of wild type and *clh-1* knockout mutants grown on control plates and on plates supplemented with 150 mM KHCO_3_ (Johnson *et al.*, 2020, Singhvi *et al.*, 2016, Fernandez-Abascal *et al.*, 2021) (Fig. 1B-C). pHlourin fluorescence intensity is proportional to the alkalinity of the cell, thus, higher fluorescence indicates that the intracellular environment is at a higher (more alkaline) pH. First, we found that AMsh glia of *clh-1* knockout animals grown on control plates had on average a fluorescence intensity twice as high as in wild type (Fig. 1B-C). This result suggests that efflux of HCO_3_^–^ from the AMsh glia is reduced in *clh-1* knockout as compared to wild type. Second, AMsh glia pHlourin fluorescence of wild type animals grown on HCO_3_^– ^enriched plates was like the fluorescence intensity seen in animals grown on control plates (Fig. 1B-C). This result indicates that in wild type AMsh glia an efficient HCO_3_^–^ extrusion mechanism keeps the pH relatively stable despite the challenge with high concentration of HCO_3_^–^. On the contrary though, the AMsh glia of *clh-1* knockout animals grown on HCO_3_^–^ enriched plates had a fluorescence intensity that on average was one and half times higher than the fluorescence intensity of these cells in animals grown on control plates (Fig. 1B-C). This result confirms that HCO_3_^–^ extrusion from AMsh glia is severely impaired in *clh-1* knockout. These data also suggest that when challenged with 150 mM HCO_3_^–^ AMsh transport HCO_3_^– ^inside the cell in a CLH-1-independent manner. A CLH-1-independent HCO_3_^–^ transport into AMsh glia was previously suggested by pH imaging of these cells under acid load challenge (Grant *et al.*, 2015).

How do these results reconcile with our previous report (Grant *et al.*, 2015)? The directionality of HCO_3_^–^ flux through a channel depends on the driving force for the ion and the membrane potential. In our previous work that showed CLH-1-mediated HCO_3_^– ^influx at baseline, pH imaging experiments were conducted on AMsh bathed in Hepes buffer and subsequently perfused with 20 mM HCO_3_^–^. Under those conditions, HCO_3_^–^ was initially depleted from the intracellular milieu, resulting in an electrochemical gradient for HCO_3_^– ^favoring inward current at negative resting membrane potential which was supported by our results. In the experiments reported here, growth in NGM control plates is expected to result in a physiological concentration of HCO_3_^– ^of ~20 mM both inside and outside the AMsh cells (Harpur, 1974). Thus, at a negative resting membrane potential, HCO_3_^– ^current is expected to be outward, which is supported by our results showing that *clh-1* knockout leads to accumulation of HCO_3_^–^ in the cell (Fig. 1C). When worms are grown in high HCO_3_^–^ the reversal potential for this ion will tend to become more negative but it seems to remain more positive than the resting membrane potential, thus, continuing to favor HCO_3_^– ^efflux through CLH-1. If *clh-1* is knocked out, then efflux is impaired and accumulation of HCO_3_^–^ is favored. Interestingly, we noticed that growth on high K^+^ (deriving from the dissociation of KHCO_3_) does not seem to dramatically change the resting potential of the cell which appears to remain still more negative than the predicted reversal potential for HCO_3_^– ^under these conditions (-47 mV). We note though that the exact concentration of K^+^ or HCO_3_^–^ in worms grown on KHCO_3_ plates is not known and may be different between the two ions.

To conclude, we propose that CLH-1 might regulate the pH outside AMsh glia, in particular in the microenvironment around the dendrites of the amphid sensory neurons, and that this might result in regulation of neuronal output. This CLH-1-dependent mechanism of regulation of neuronal function might be in addition to providing Cl^–^ ions for GABA signaling in nose touch avoidance (Fernandez-Abascal *et al.*, 2021). Future imaging and behavioral experiments using an extracellular pH sensor and CLH-1 mutants with altered Cl^–^ and HCO_3_^–^ permeability will test this hypothesis.

## Methods

***C. elegans* growth and maintenance:** Animals were grown at 20°C on standard nematode medium (NGM) seeded with *Escherichia coli* (strain OP50). Experiments were performed on young adult hermaphrodites.

**Plate supplementation:** NGM plates were prepared as usual and a solution containing autoclaved KHCO_3 _was added to the media right before pouring into plates, to a final concentration of 150 mM. Worms were grown in supplemented plates from egg to young adults.

**Worm bleaching**: Gravid adults were collected from plates using M9 buffer and transferred into tubes for centrifugation (5 mins at 4300 rpm). Next, the pelleted worms were treated with a solution containing 0.1 M NaOH and 22.7% commercial bleach for 5-10 minutes. When about 90% of the eggs were released, they were washed with M9 twice. Eggs were then seeded onto control and supplemented plates.

***C. elegans* strains**: The following strains from (Grant *et al.*, 2015) were used: BLC44 *Ex405[pT02B11.3::pGM87]* and BLC295 *clh-1(ok658); Ex405[pT02B11.3::pGM87]*. The BLC295 strain was obtained by crossing *clh-1(ok658)* with BLC44. The *clh-1(ok658)* strain used in the cross described was RB833.

**Fluorescence microscopy:** Animals were immobilized with 100 mM NaN_3_ on 2% agarose pads. An Evos FL Auto 2 Imaging System (Invitrogen) equipped with a 40x objective (Olympus) was used to acquire the images of the AMsh cell body. An average of 6 stacks (0.6 µm each) on the Z axis were acquired per cell using the Evos FL Auto 2 software. Stacks were then processed using the “Z project” plugin (maximum intensity as projection type) from Fiji (ImageJ software). The resulting images were used to quantify the pixel intensity of the AMsh glia as an averaged intensity per area. Data were normalized to N2 in NGM conditions.

## References

[R1] Bacaj T, Tevlin M, Lu Y, Shaham S (2008). Glia are essential for sensory organ function in C. elegans.. Science.

[R2] Chesler M (2003). Regulation and modulation of pH in the brain.. Physiol Rev.

[R3] Fernandez-Abascal J, Johnson CK, Graziano B, Wang L, Encalada N, Bianchi L (2021). A glial ClC Cl^-^ channel mediates nose touch responses in C. elegans.. Neuron.

[R4] Grant J, Matthewman C, Bianchi L (2015). A Novel Mechanism of pH Buffering in C. elegans Glia: Bicarbonate Transport via the Voltage-Gated ClC Cl- Channel CLH-1.. J Neurosci.

[R5] Harpur RP (1974). Haemolymph gases and buffers in Ascaris lumbricoides.. Comp Biochem Physiol A Comp Physiol.

[R6] Johnson CK, Fernandez-Abascal J, Wang Y, Wang L, Bianchi L (2020). The Na^+^-K^+^-ATPase is needed in glia of touch receptors for responses to touch in *C. elegans*.. J Neurophysiol.

[R7] Oikonomou G, Perens EA, Lu Y, Watanabe S, Jorgensen EM, Shaham S (2011). Opposing activities of LIT-1/NLK and DAF-6/patched-related direct sensory compartment morphogenesis in C. elegans.. PLoS Biol.

[R8] Raiders S, Black EC, Bae A, MacFarlane S, Klein M, Shaham S, Singhvi A (2021). Glia actively sculpt sensory neurons by controlled phagocytosis to tune animal behavior.. Elife.

[R9] Razzauti A, Laurent P (2021). Ectocytosis prevents accumulation of ciliary cargo in *C. elegans* sensory neurons.. Elife.

[R10] Singhvi A, Liu B, Friedman CJ, Fong J, Lu Y, Huang XY, Shaham S (2016). A Glial K/Cl Transporter Controls Neuronal Receptive Ending Shape by Chloride Inhibition of an rGC.. Cell.

[R11] Wallace SW, Singhvi A, Liang Y, Lu Y, Shaham S (2016). PROS-1/Prospero Is a Major Regulator of the Glia-Specific Secretome Controlling Sensory-Neuron Shape and Function in C. elegans.. Cell Rep.

[R12] Wang Y, D'Urso G, Bianchi L (2011). Knockout of glial channel ACD-1 exacerbates sensory deficits in a C. elegans mutant by regulating calcium levels of sensory neurons.. J Neurophysiol.

[R13] Wang Y, Apicella A Jr, Lee SK, Ezcurra M, Slone RD, Goldmit M, Schafer WR, Shaham S, Driscoll M, Bianchi L (2008). A glial DEG/ENaC channel functions with neuronal channel DEG-1 to mediate specific sensory functions in C. elegans.. EMBO J.

